# Mutation-resolved single-cell transcriptomics reveals enhanced **β**-amyloid clearance in TET2-mutant human monocytes

**DOI:** 10.1172/jci.insight.208804

**Published:** 2026-08-24

**Authors:** Xiao Yang, Sameen Fatima, Salvador Sampere-Birlanga, Akshay Ware, Mariana Shumliakivska, Lukas Zanders, Srisurekha Radhakrishnan, Guillermo Luxán, David John, Stefan Günther, Silvia Mas-Peiro, Stefanie Dimmeler, Andreas M. Zeiher, Wesley T. Abplanalp

**Affiliations:** 1 Institute for Cardiovascular Regeneration, Goethe University Frankfurt, Frankfurt, Germany.; 2 German Center for Cardiovascular Research, Partner Site RheinMain, Germany.; 3 Cardiopulmonary Institute, Frankfurt, Germany.; 4 Max Planck Institute for Heart and Lung Research, Bad Nauheim, Germany.

**Keywords:** Aging, Immunology, Neuroscience, Alzheimer disease

## Abstract

TET2-driven clonal hematopoiesis has been associated with reduced Alzheimer’s disease risk, but mechanisms in humans remain unclear. Using mutation-resolved single-cell transcriptomics, we distinguished TET2-mutant and wild-type monocytes within the same aged individuals and identified enrichment of phagocytosis and complement programs in mutant cells. TET2 silencing in human monocytes and macrophages recapitulated this phenotype and enhanced β-amyloid uptake, indicating mutation-specific bias of innate immunity toward aggregate clearance.

**To the Editor: ***TET2*-driven clonal hematopoiesis has been associated with reduced Alzheimer’s disease risk, but its mechanisms remain unclear. Using high-throughput mutation-resolved single-cell transcriptomics, we directly distinguished *TET2*-mutant and WT monocytes within the same aged individuals and identified enrichment of phagocytosis and complement programs in mutant cells. *TET2* silencing in human monocytes and macrophages recapitulated this phenotype and enhanced β-amyloid uptake, indicating mutation-specific bias of innate immunity toward aggregate clearance.

Age-related clonal hematopoiesis of indeterminate potential (CHIP) arises from expansion of hematopoietic clones carrying somatic mutations such as *TET2* (1). Although CHIP promotes cardiovascular disease, epidemiological studies have paradoxically linked *TET2*-mutant CHIP to reduced Alzheimer’s disease incidence, suggesting mutation-specific effects on aging immunity (2, 3). Experimental mouse models indicate enhanced β-amyloid phagocytosis after *TET2* loss (4), but whether *TET2*-mutant clonal hematopoiesis confers cell-intrinsic functional biases to human innate immune cells remained unclear. This requires mutation-resolved single-cell analysis distinguishing mutant and nonmutant cells within the same individuals.

To address this question, we applied high-throughput mutation-resolved single-cell transcriptomics to PBMCs from 8 elderly individuals (77–91 years), including 3 without detectable CHIP (No-CHIP; *n* = 2 females) and 5 carrying *TET2* mutations with variant allele frequencies (2.6%–37.4%; *n* = 3 females). PBMCs underwent single-cell RNA-seq, yielding 73,980 high-quality transcriptomes after filtering. Unsupervised clustering and UMAP projection identified canonical immune populations with similar distributions across donors (Figure 1A). Immune cell–type proportions did not differ between *TET2*-CHIP and No-CHIP donors (*P* = 0.2). A phagocytosis module score across PBMCs identified monocytes as the dominant phagocytic population, with minimal activity in lymphoid cells (Figure 1B).

We next examined the monocyte compartment (12,527 cells), where phagocytosis module scores were significantly higher in aged *TET2*-CHIP individuals compared with those in the No-CHIP control group (Figure 1C). To resolve this difference at the clonal level, we applied mutation-resolved single-cell RNA-seq (MutDetect-seq) to assign mutation status (Figure 1D) (5). Phagocytosis scores were elevated in *TET2*-mutant versus WT monocytes from the same individuals, indicating a cell-intrinsic effect (Figure 1E).

Consistent with these observations, differential gene expression and pathway analyses revealed enrichment of complement, phagosome maturation, and ROS-related pathways in *TET2*-mutant monocytes (Supplemental Figure 1A). Increased phagocytosis module scores were also observed in WT monocytes from *TET2*-CHIP donors compared with WT monocytes from No-CHIP donors (Supplemental Figure 1B). Upregulated genes included *CD14*, *CLEC7A*, *COLEC12*, *VSIG4*, and *CCL2* (Supplemental Figure 1C), supporting coordinated activation of aggregate recognition and complement-mediated clearance programs.

To determine whether *TET2* loss directly drives this phenotype, we silenced *TET2* in human primary monocyte-derived macrophages. RNA-seq profiling of primary monocyte-derived macrophages treated with small interfering RNA targeting *TET2* (si*TET2*)-treated cells recapitulated the phagocytosis and complement gene induction observed in *TET2*-CHIP monocytes (Supplemental Figure 1D). Functionally, *TET2* silencing increased β-amyloid uptake by 1.17-fold relative to control (*P* = 0.018; Figure 1, F–H, and Supplemental Figure 1E), confirming a reproducible, cell-intrinsic enhancement of phagocytosis.

Together, these results demonstrate that *TET2* mutations bias circulating human monocytes toward a phagocytosis-competent state characterized by upregulation of complement and cytoskeletal effectors. This transcriptional and functional phenotype was recapitulated by *TET2* silencing in two independent human macrophage models, supporting that *TET2* constrains amyloid-responsive phagocytic activation. The results extend prior murine observations to humans and support the hypothesis that clonal hematopoiesis can modulate systemic innate immunity in a manner that may confer protection from Alzheimer’s disease (2, 4).

While prior studies demonstrated CNS recruitment and enhanced phagocytosis of *TET2*-mutant myeloid cells in mouse transplantation models, our data established this phagocytic bias in endogenous human *TET2*-mutant clones. Together, these data show that *TET2* mutations bias peripheral monocytes toward a complement-enriched phagocytic phenotype, consistent with murine and iPSC data (4). These transcriptional programs may also involve broader phagosomal processing and ROS-associated pathways, potentially influencing the intracellular handling of β-amyloid aggregates. Notably, this effect is demonstrated here for what we believe to be the first time in endogenous human *TET2*-mutant clones, enabled by mutation-resolved single-cell transcriptomics, rather than transplantation or in vitro models. Such functional reprogramming may reflect the role of *TET2* in innate immune regulation. Loss of *TET2* activity has been shown to promote inflammatory myeloid programs, which may contribute to the enhanced complement-associated and phagocytic signatures observed in *TET2*-mutant monocytes.

Prior work showed that *TET2*-mutant, but not *DNMT3A*-mutant, myeloid cells are recruited into the aging brain, adopt microglia-like states, and enhance β-amyloid clearance in mouse transplantation and iPSC-microglia models, partly through *CCL2*/*CCR2*-mediated chemotaxis and elevated phagocytosis (4). Using mutation-resolved single-cell transcriptomics, we show for what we believe to be the first time that this phagocytic bias is intrinsic to endogenous *TET2*-mutant clones in aged humans, evidenced by upregulation of *CLEC7A*, *COLEC12*, and *VSIG4* and a higher phagocytosis module score in mutant versus WT monocytes from the same individuals.

These data indicate that *TET2*-driven clonal hematopoiesis does not uniformly promote proinflammatory phenotypes, as described in cardiovascular disease (3), but instead biases peripheral myeloid cells toward enhanced aggregate clearance — a mutation-specific remodeling of innate immunity that may contribute to the reduced Alzheimer’s disease risk observed in CHIP carriers, particularly those with high-variant allele frequencies (2). This refines the concept of CHIP as a functionally heterogeneous, rather than uniformly deleterious, state (6). Further work integrating immune phenotyping, lysosomal assays, brain-imaging correlates, and epigenomics will clarify how peripheral *TET2*-mutant clones interact with CNS compartments to shape neurodegenerative trajectories.

For detailed methods, information regarding sex as a biological variable, statistics, study approval, data availability, author contributions, and acknowledgments, see the Supplemental Methods. For detailed demographic and comorbidity characteristics of the study participants, see Supplemental Table 1. 

## Funding support

- Dr. Rolf M. Schwiete Foundation (to SD).
- German Research Foundation (SFB834/SFB1531, projects B1 and S2, HERZBLUT Forschergruppe A02, to SD, AMZ, SMP, and WTA).
- European Research Council (CHIP-AVS, Neuroheart, to AMZ and SD).
- German Center for Cardiovascular Research (to SD, AMZ, and WTA).

## Conflict of interest

The authors have declared that no conflict of interest exists.

## Supplementary Material

Supplemental data

Supporting data values

## Figures and Tables

**Figure 1:**
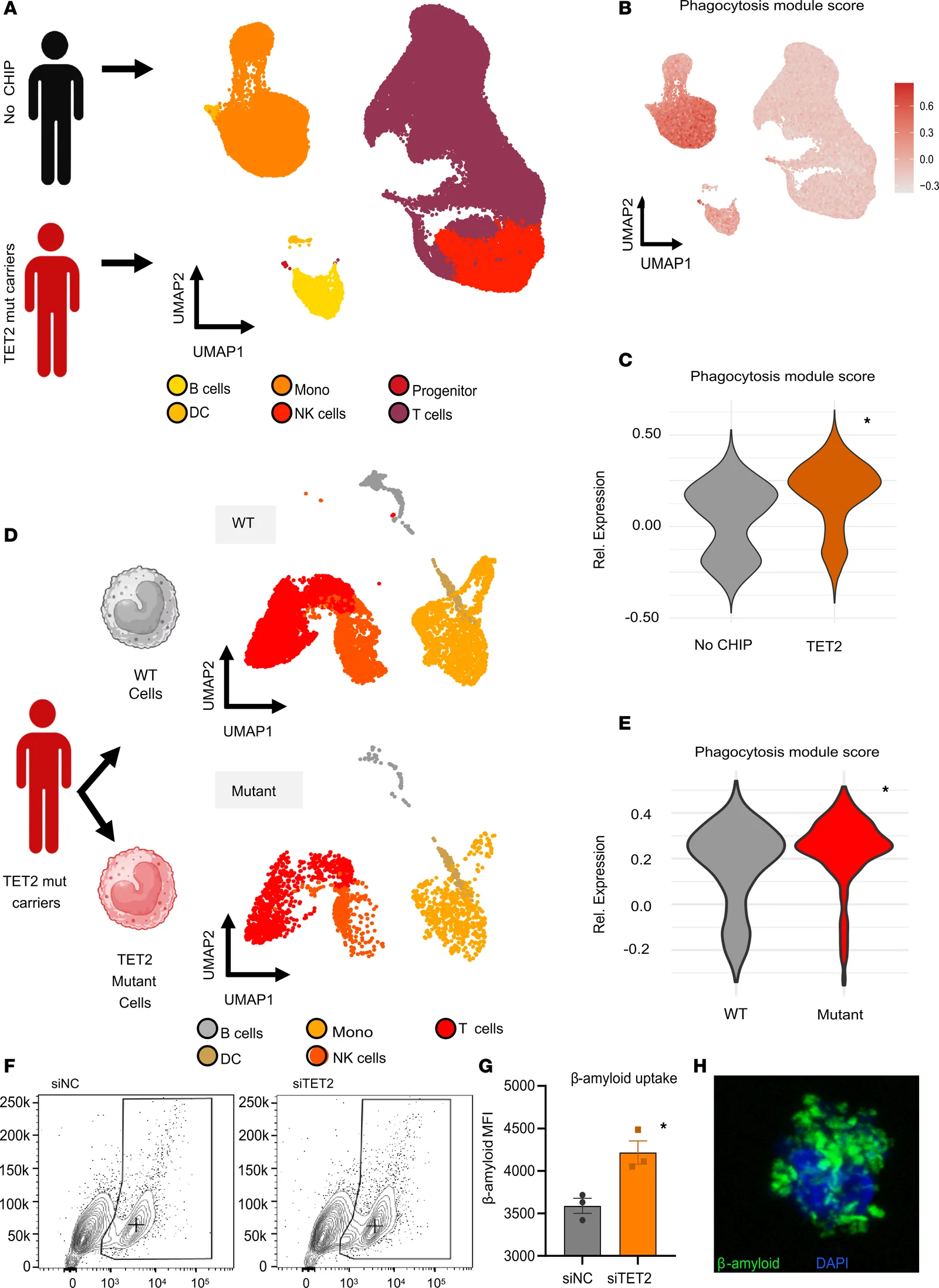
TET2-mutant carriers and cells exhibit enhanced phagocytic signatures in aged individuals. (**A**) UMAP of PBMCs from aged individuals with or without *TET2*-CHIP, showing major immune populations. (**B**) Phagocytosis module scores across immune cell types. (**C**) Phagocytosis module scores in monocytes from No-CHIP controls and *TET2*-mutant individuals. (**D**) Mutation-resolved single-cell RNA-seq identifying WT and *TET2*-mutant cells. (**E**) Phagocytosis module scores in WT versus *TET2*-mutant monocytes. (**F** and **G**) Representative flow cytometry plots and quantification of β-amyloid uptake in si*TET2* and control monocytes. (**H**) Fluorescence microscopy of β-amyloid uptake in monocytes. Statistics: Wilcoxon rank-sum test (**C** and **E**); paired 2-tailed Student’s *t* test (**G**). Data are means ± SEM. *P < 0.05.

## Version 1 08/24/2026

Electronic publication
