# Supplementary material for: Mutation-resolved single-cell transcriptomics reveals enhanced β-amyloid clearance in TET2-mutant human monocytes
Source: JCI Insight. 2026 Aug 24;11(16):e208804. doi: 10.1172/jci.insight.208804 (PMC13502165; doi:10.1172/jci.insight.208804)
Supplement: Supplementary file 1 [file jciinsight-11-208804-s258.pdf]

Supplemental Methods

Sex as a biological variable

Both female and male participants were included in this study. The cohort comprised three individuals without detectable CHIP (2 females and 1 male) and five individuals carrying TET2 mutations (3 females and 2 males). Sex was not considered as a biological variable in the study design, and sex-stratified analyses were not performed because of the limited sample size. Therefore, the findings reflect the overall study population. Future studies with larger cohorts will be required to determine whether the observed transcriptional and phagocytic phenotypes differ between sexes.

Study Design and Experimental Procedures

Peripheral blood mononuclear cells (PBMCs) were obtained from elderly individuals with or without TET2-mutant clonal hematopoiesis and processed for single-cell RNA sequencing using the 10x Genomics platform. Data was analyzed using Cell Ranger and Seurat to identify immune cell populations and compute phagocytosis-related module scores. TET2 mutations were identified by targeted deep sequencing of peripheral blood DNA. For functional validation, primary human monocyte-derived macrophages were generated, subjected to *TET2* knockdown using siRNA, and analyzed by RNA sequencing and β-amyloid uptake assays. Detailed methods are provided in the supplemental material.

Study population and blood collection

Peripheral blood samples were obtained from elderly individuals who had undergone successful transcatheter aortic valve implantation (TAVI) and were enrolled in a prospective observational cohort at Goethe University Hospital. Participants were screened for clonal hematopoiesis driver mutations using targeted DNA sequencing of peripheral blood. Individuals carrying somatic TET2 mutations were classified as TET2-CHIP, whereas mutation-negative individuals served as controls.

Blood was collected between 10:00 and 12:00 to minimize circadian variability and processed within 2 hours of collection. PBMCs were isolated by density gradient centrifugation, washed in PBS, and either processed immediately for single-cell RNA sequencing or cryopreserved for batch analysis.

Exclusion criteria included active infection, recent major surgery (<3 months), known hematologic malignancy, or immunosuppressive therapy. Demographic characteristics and relevant comorbidities of study participants are provided in Supplementary Table S1.

Single-cell RNA sequencing and analysis

Single-cell suspensions were loaded onto a Chromium Controller (10x Genomics) targeting recovery of approximately 5,000–10,000 cells per sample. Libraries were prepared using the Chromium Single Cell 3′ Gene Expression Kit (v3 or v3.1; 10x Genomics) according to the manufacturer’s instructions. Briefly, cells were partitioned into nanoliter droplets containing barcoded gel beads to generate gel bead-in-emulsions (GEMs). Following reverse transcription, emulsions were broken and barcoded cDNA

was purified using silane magnetic beads. cDNA was amplified by PCR, fragmented, end-repaired, A-tailed, adaptor-ligated, and sample-indexed.

Library quality and fragment size distribution were assessed using an Agilent Bioanalyzer High Sensitivity DNA chip, and concentrations were determined by fluorometric quantification. Indexed libraries were pooled equimolarly and sequenced on an Illumina NovaSeq 6000 platform using paired-end sequencing (read 1: 26 bp; read 2: 98 bp; index reads: 8 bp). Sequencing depth targeted a minimum of 50,000 reads per cell.

Raw base call files were converted to FASTQ format and processed using Cell Ranger (10x Genomics) with alignment to the human reference genome GRCh38. Downstream analyses were conducted in R using Seurat (version 4). Cells with fewer than 200 detected genes, >10–15% mitochondrial transcripts, or abnormally high UMI counts indicative of doublets were excluded. Gene expression values were log-normalized using a scaling factor of 10,000. Highly variable genes were identified for principal component analysis (PCA), and the top principal components were used for graph-based clustering and Uniform Manifold Approximation and Projection (UMAP) visualization.

Cell types were annotated based on canonical marker genes for major immune populations. Monocyte subsets were defined according to CD14 and FCGR3A expression into classical, intermediate, and non-classical populations. Differential gene expression analyses between TET2-CHIP and control groups were performed using a Wilcoxon rank-sum test with false discovery rate correction. Gene ontology and pathway enrichment analyses were conducted using Metascape.

Phagocytosis module scoring

A curated gene set associated with phagocytosis and complement activation was compiled from published datasets and Gene Ontology annotations. Module scores were calculated using the AddModuleScore function in Seurat.

Variant detection for clonal hematopoiesis

Targeted deep sequencing of CHIP-associated genes was performed on genomic DNA extracted from peripheral blood leukocytes. Reads were aligned to the reference genome, and variants were called and annotated using standard pipelines. Common germline polymorphisms were excluded using population databases. Somatic mutations were defined based on variant allele frequency thresholds and absence in matched controls where available.

THP1 monocyte-derived macrophage differentiation and TET2 silencing

THP-1 human monocytic cells (ATCC TIB-202™, American Type Culture Collection, Manassas, VA, USA) were differentiated into macrophages and were not further polarized toward canonical M1 or M2 macrophage phenotypes prior to experimentation. TET2 knockdown was achieved using siRNA transfection, and knockdown efficiency was confirmed prior to downstream analyses.

Bulk RNA sequencing

Total RNA was extracted and subjected to bulk RNA sequencing. Reads were processed, aligned to the human genome, and quantified using standard pipelines.

β-amyloid uptake assay and flow cytometry

Phagocytic uptake of fluorescently labeled β-amyloid was assessed in monocytes or macrophages. Cells were incubated with HiLyte™ Fluor 488-labeled β-amyloid (1–40) peptide (Biotrend, Cat# AS-60491-01). Following incubation and washing, dead cells were excluded using Zombie UV™ Fixable Viability Kit (BioLegend, Cat# 423107). Flow-cytometric analyses were performed by sequential gating on cells, singlets, and live cells prior to quantification of β-amyloid uptake as mean fluorescence intensity or percentage of positive cells relative to controls.

Statistics

Statistical analyses were performed using R and GraphPad Prism. Data are presented as mean ± SEM unless otherwise indicated. For single-cell RNA-seq analyses, differential gene expression and group comparisons were performed using the Wilcoxon rank-sum test implemented in the Seurat package. Multiple testing correction was performed using the false discovery rate (FDR) method. For primary monocyte-derived macrophage experiments, paired two-tailed Student's t tests were used. A two-sided P value < 0.05 was considered statistically significant.

Study approval

The study was conducted in accordance with the Declaration of Helsinki and approved by the Ethics Committee of the Faculty of Medicine, Goethe University Frankfurt (Frankfurt, Germany). Written informed consent was obtained from all participants prior to enrollment.

Data availability

All raw and processed scRNA-seq data have been deposited in the public archive ArrayExpress (accession number: E-MTAB-15820). All raw bulk RNA-seq data have been deposited in the

public archive ArrayExpress (accession number: E-MTAB-17157). Supporting Data Values are provided as a separate Excel file containing the numerical values underlying all graphs presented in the figures. Additional data and analytical code supporting this study are available from the corresponding author upon reasonable request.

Author contributions

XY, SF, SSB, AW, MS, LZ, SR, DJ, SMP, SD, AMZ, and WTA wrote the manuscript. XY, SF, SSB, MS, LZ, SMP, SD, AMZ, and WTA designed the research studies. XY, SF, SSB, LX, AW, MS, LZ, SR, DJ, and WTA conducted experiments. XY, SF, SSB, MS, SR, GL and WTA acquired data. XY, SF, SSB, AW, MS, LZ, SR, DJ, SG and WTA analyzed data. XY, SF, SSB, MS, LZ, SR, DJ, SMP, SD, and WTA provided reagents.

Acknowledgements

The authors thank Bianca Schuhmacher for her technical support.

Supplemental Figure 1

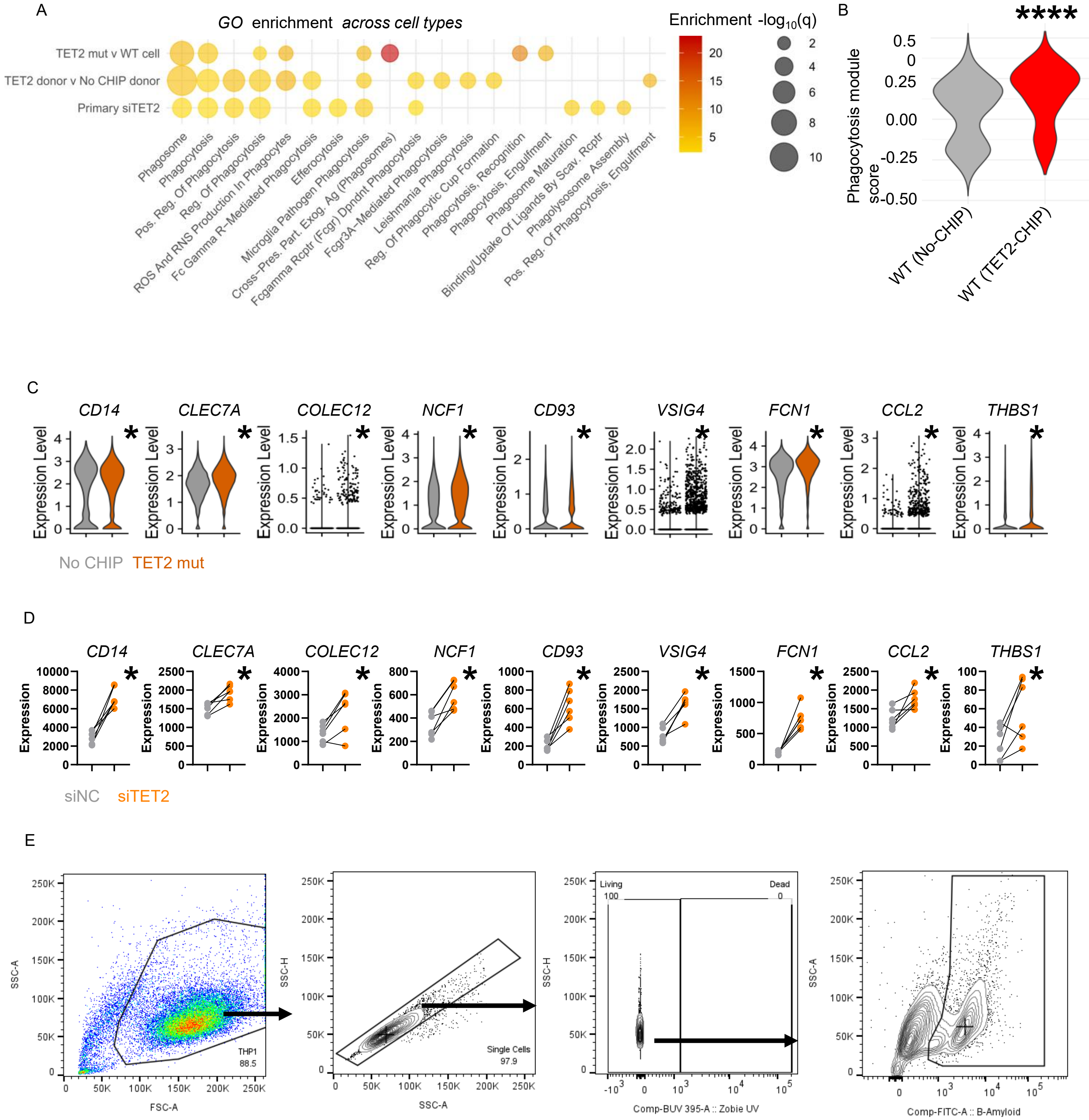

Supplemental Figure 1. Phagocytosis-associated gene expression and pathway enrichment in *TET2* loss-of-function models and flow cytometry gating strategy.
A. Gene ontology terms from upregulated genes across three *TET2* loss-of-function models show enhanced phagocytosis pathways relative to wild-type cells or appropriate controls. B. Phagocytosis module scores were also increased in WT monocytes from TET2-CHIP donors compared with WT monocytes from No-CHIP donors. C–D. Phagocytosis-associated genes upregulated in *TET2* loss-of-function analyses for (C) human TET2-mutant carriers and (D) primary monocyte-derived macrophage *TET2* silencing model. E. Flow cytometry gating strategy for β-amyloid flow cytometry analysis. Statistics: Wilcoxon rank-sum test (B, ****P < 0.0001); Seurat FindMarkers with FDR correction (C–D, *P < 0.05).

Supplementary Table S1. Comorbidities of study participants included in the scRNA-seq analysis.

Characteristic	No-CHIP (n = 3)	TET2-CHIP (n = 5)
**Demographics and comorbidities**		
Age	84 (83-87)	86 (77-91)
eGFR	62 (46-71.4)	59.9 (32.9-75.4)
Cerebrovascular disease	0 (0%)	0 (0%)
Peripheral arterial occlusive disease	0 (0%)	0 (0%)
COPD	0 (0%)	0 (0%)
Diabetes	0 (0%)	1 (20%)
Hypertension	3 (100%)	5 (100%)
Previous MI	0 (0%)	0 (0%)
Previous stroke	0 (0%)	2 (40%)
Previous TIA	0 (0%)	0 (0%)
Previous PCI	1 (33.3%)	2 (40%)
CAD	1 (33.3%)	2 (40%)
Atrial fibrillation	2 (66.7%)	4 (80%)
